# Topical Application of Ochratoxin A Causes DNA Damage and Tumor Initiation in Mouse Skin

**DOI:** 10.1371/journal.pone.0047280

**Published:** 2012-10-10

**Authors:** Rahul Kumar, Kausar M. Ansari, Bhushan P. Chaudhari, Alok Dhawan, Premendra D. Dwivedi, Swatantra K. Jain, Mukul Das

**Affiliations:** 1 Food, Drug and Chemical Toxicology Group, CSIR-Indian Institute of Toxicology Research (CSIR-IITR), Lucknow, Uttar Pradesh, India; 2 Department of Biotechnology, Faculty of Science, Jamia Hamdard (Hamdard University), New Delhi, India; 3 Pathology Laboratory, CSIR-Indian Institute of Toxicology Research (CSIR-IITR), Lucknow, Uttar Pradesh, India; 4 Nanotoxicology Group, CSIR-Indian Institute of Toxicology Research (CSIR-IITR), Lucknow, Uttar Pradesh, India; University of Maryland School of Medicine, United States of America

## Abstract

Skin cancer is one of the most common forms of cancer and 2–3 million new cases are being diagnosed globally each year. Along with UV rays, environmental pollutants/chemicals including mycotoxins, contaminants of various foods and feed stuffs, could be one of the aetiological factors of skin cancer. In the present study, we evaluated the DNA damaging potential and dermal carcinogenicity of a mycotoxin, ochratoxin A (OTA), with the rationale that dermal exposure to OTA in workers may occur during their involvement in pre and post harvest stages of agriculture. A single topical application of OTA (20–80 µg/mouse) resulted in significant DNA damage along with elevated γ-H2AX level in skin. Alteration in oxidative stress markers such as lipid peroxidation, protein carbonyl, glutathione content and antioxidant enzymes was observed in a dose (20–80 µg/mouse) and time-dependent (12–72 h) manner. The oxidative stress was further emphasized by the suppression of Nrf2 translocation to nucleus following a single topical application of OTA (80 µg/mouse) after 24 h. OTA (80 µg/mouse) application for 12–72 h caused significant enhancement in- (a) reactive oxygen species generation, (b) activation of ERK1/2, p38 and JNK MAPKs, (c) cell cycle arrest at G0/G1 phase (37–67%), (d) induction of apoptosis (2.0–11.0 fold), (e) expression of p53, p21/waf1, (f) Bax/Bcl-2 ratio, (g) cytochrome *c* level, (h) activities of caspase 9 (1.2–1.8 fold) and 3 (1.7–2.2 fold) as well as poly ADP ribose polymerase cleavage. In a two-stage mouse skin tumorigenesis protocol, it was observed that a single topical application of OTA (80 µg/mouse) followed by twice weekly application of 12-O-tetradecanoylphorbol-13-acetate for 24 week leads to tumor formation. These results suggest that OTA has skin tumor initiating property which may be related to oxidative stress, MAPKs signaling and DNA damage.

## Introduction

Skin being the largest organ of human body, is exposed to sunlight, air pollutants and other environmental factors which may lead to skin cancer. According to World Health Organization (WHO), incidence of non-melanoma skin cancer has been on an increase and almost 2 to 3 million new cases are being diagnosed annually [1]. Although, ultra violet (UV) rays from sunlight are considered as the main cause of skin cancer; nonetheless, dermal exposure to other potential chemical carcinogens including mycotoxins can not be ruled out and their toxicity needs to be evaluated.

Mycotoxins are fungal secondary metabolites, commonly found in food crops and considered to be unavoidable contaminants worldwide due to the widespread nature of fungi in the environment. Ochratoxin A (OTA), a mycotoxin produced by several *Aspergillus* and *Penicillium* species, is a common contaminant in wheat, barley, grapes, coffee, spices, etc. and affects the health of humans as well as livestock [2]. Earlier studies have shown that kidney is the main target organ for OTA toxicity apart from developing neurons, immune system and liver [3]. OTA has also been reported to trigger nephropathy as well as renal adenoma formation in various farm animals and rodents [4]. Further, OTA has been suspected as a possible aetiological agent in human Balkan Endemic Nephropathy (BEN) causing renal tract tumors in the affected population of Balkan region [5]. However, available epidemiological information is insufficient to assess the direct correlation of OTA consumption through food with human health risk [6], [7]. Hence, International Agency for Research on Cancer (IARC) has classified OTA as a group 2B carcinogen [8]. It has been widely acknowledged that the risk assessment to OTA exposure requires more of animal data that would significantly help in the elucidation of the mechanism(s) of carcinogenicity [6], [7]. Although, OTA has been shown to be genotoxic which may play an important role in OTA carcinogenicity, the actual molecular mechanism of action either through DNA adduct formation or through epigenetic pathway(s) is still debatable [6], [9]. Several epigenetic mechanisms including protein synthesis inhibition, oxidative stress, alterations of cell signaling pathways and apoptosis in cell lines have been reported for OTA carcinogenicity [6], [7], [9].

WHO has highlighted the need for toxicological studies through dermal exposure, as limited data is available regarding epidermal exposure risk to mycotoxins [10]. This is an important aspect from the point of view of developing countries in tropics including India where manual labour is employed without any protective measure during pre- and post-harvest stages of agriculture, thereby causing a probable exposure risk through dermal route. In this regard our prior studies have revealed that dermal exposure to some mycotoxins viz aflatoxin B1 (AFB1), patulin and citrinin caused skin toxicity including tumor formation [11]–[14]. However, no such data is available for OTA. Therefore, the aim of the present study was to evaluate the skin tumorigenic property of OTA using a well established two-stage mouse skin tumorigenesis protocol and to understand the cellular and molecular events leading to OTA mediated dermal tumorigenicity.

## Materials and Methods

### Chemicals

Ochratoxin A (OTA), 7,12-dimethylbenz[α]anthracene (DMBA), 12-O-tetradecanoylphorbol-13-acetate (TPA), 2′,7′-dichlorodihydrofluorescein diacetate (H_2_DCFDA), dithiothreteiol (DTT), phenylmethylsulphonyl fluoride (PMSF), 2-mercaptoethanol (BME), propidium iodide (PI), RNase A, normal melting agarose (NMA), low melting point agarose (LMPA), ethidium bromide (EtBr), ethylenediamine tetraacetic acid (EDTA) disodium salt, Tris buffer, triton X-100, bovine serum albumin (BSA), and anti-γ-H2AX (Ser^139^) were obtained from Sigma Chemicals Co. (St. Louis, MO). Anti-p53, anti-Bcl-2, anti-cytochrome c, anti-poly ADP ribose polymerase (PARP), anti-β-actin, anti-p- Extracellular signal-regulated kinases (ERK1/2) (Thr^202^/Tyr^204^), anti-Nuclear Factor-erythroid 2 (Nrf2), anti-ERK1/2, anti-p38, anti-c-jun N-terminal kinase (JNK) and blotto (non fat dry milk) were purchased from Santa Cruz Biotechnology (Santa Cruz, CA). Anti-p21/waf1, anti-p-p38 (Thr^180^/Tyr^182^), anti-p-JNK (Thr^183^/Tyr^185^) and anti-Bax were procured from Cell Signaling Technology (Beverly, MA). Annexin V-PI Apoptosis Detection Kit and specific substrates for Caspase 3, 8 and 9 were the products of BD Pharmingen (San Jose, CA). Halt Protease and Phosphatase Inhibitor Cocktail was procured from Thermo Scientific (Rockford, IL). All other chemicals used were of the highest purity commercially available.

### Animals and Ethics Statement

Six to seven week old female Swiss albino mice (20±3 g) derived from the animal breeding colony of CSIR-Indian Institute of Toxicology Research (CSIR-IITR), Lucknow, were acclimatized under standard laboratory conditions and given a commercial pellet diet (Ashirwad Industries, Chandigarh, India) and water *ad libitum*. Animals were housed in plastic cages having rice husk as bedding and maintained in a controlled atmosphere of 12 h dark/light cycle, 22±2°C temperature and 50–60% humidity as per rules laid down by Animal Welfare Committee of CSIR-IITR. All the experiments involving animals were approved by the Institutional Animal Ethics Committee (IAEC), CSIR-IITR, Lucknow vide approval # ITRC/IAEC/26/2008. The mice were shaved with an electric clipper (Oster, WI, USA) one week prior to the beginning of the experiment. Mice showing no signs of hair growth were used for further experiments. Animals were sacrificed by cervical dislocation with minimal suffering as per CSIR-IITR guidelines.

### Alkaline Comet Assay and γ-H2AX Western Blot Analysis

The animals were distributed randomly into four groups, having five mice per group. The mice of 1^st^ group received a single topical application of 0.2 ml acetone to serve as control, while animals of 2^nd^, 3^rd^ and 4^th^ group received a single topical application of 20, 40 and 80 µg OTA in 0.2 ml acetone, respectively. Following 24 h of treatment, mice were sacrificed and ∼2 cm^2^ of the exposed skin was excised. Single cell suspension from mouse skin was prepared as described earlier [12]. DNA damage in mouse skin cells was evaluated by the alkaline comet assay as described previously [12], [15].

For western blot analysis of γ-H2AX, the animals were distributed randomly into four groups, having three mice per group. The animals were treated as described above in alkaline comet assay and whole cell extract of skin was prepared as described earlier [16] with slight modifications. Briefly, skin was homogenized in radio immunoprecipitation assay (RIPA) buffer [50 mM Tris–HCl (pH 7.5), 1% nonidet P-40, 0.25% sodium deoxycholate, 0.25% SDS, 150 mM NaCl, 1 mM EGTA, 0.2 mM PMSF] supplemented with Halt Protease and Phosphatase Inhibitor Cocktail (Thermo Scientific, Rockford, IL) using Ultra Turrax Polytron (Janke & Kunkel, IKA-Labortechnik, Staufen, Germany) followed by 60 min incubation on ice with an intermittent pulse at every 15 min. The extracts were centrifuged at 14,000 rpm for 20 min and the supernatants were stored at −80°C after protein quantification. Eighty micrograms of protein extract was resolved on 12% SDS-polyacrylamide gel and the proteins were transferred to polyvinylidene fluoride (PVDF) membranes. The blotted membrane was blocked with 5% BSA in PBS containing 0.1% Tween 20 (blocking solution), and incubated with a specific antibody against γ-H2AX at the dilution indicated by the manufacturer. The blots were further incubated with HRP-conjugated secondary antibody (Sigma Chemical Co., St. Louis, MO) and developed by ECL Western Blotting Detection Kit as described in the manufacturer’s protocol (Amersham, Fairfield, CT). The blots were stripped and reprobed with β-actin to ensure equal loading of protein.

### ROS Measurement

The animals were distributed randomly into five groups, having five mice per group. The mice of 1^st^ group received a single topical application of 0.2 ml acetone to serve as control, while animals of 2^nd^, 3^rd^, 4^th^ and 5^th^ group received a single topical application of 80 µg OTA in 0.2 ml acetone and sacrificed after 12, 24, 48 and 72 h, respectively. A ∼2 cm^2^ of the exposed skin was excised and single cell suspension was prepared as described earlier [12]. For estimation of ROS, 1×10^5^ cells were incubated in the presence of 100 µM H_2_DCFDA for 60 min at 37°C in a black 96-well plate and relative fluorescence intensity was measured in SYNERGY-HT multiwell plate reader (Bio-Tek, Winooski, VT) using KC4 software at an excitation and emission wavelengths of 485 and 520 nm, respectively.

### Oxidative Stress Markers

To study the dose dependent effect of OTA on various oxidative stress markers, the animals were distributed randomly into four groups, having five mice per group. Treatment with OTA has been described above in alkaline comet assay. For studying the time dependent effect of OTA, animals from ROS experiments were used. Fat from the dermal side of excised skin was removed by scraping and 10% w/v skin homogenate was prepared in 0.2 M phosphate buffer using Ultra Turrax Polytron. A portion of the skin homogenate was centrifuged at 10,000 *g* for 20 min at 4°C to isolate post mitochondrial supernatant. Reduced glutathione (GSH) content was assayed in the skin homogenate according to the method of Ellman [17]. Lipid peroxidation (LPO) in the skin homogenate was determined by estimating the formation of malondialdehyde (MDA) [18]. Protein carbonyl content was measured in the skin homogenate according to the method of Levine *et al.* [19]. Catalase activity was determined by the method of Sinha [20]. Glutathione reductase (GR) and glutathione S transferase (GST) activities were assayed according to the method of Moron *et al.* [21].

### Cell Cycle Analysis

The treatment schedule for cell cycle analysis was the same as described in the section “ROS measurement” and single cell suspensions were prepared as described earlier [12]. Single cell suspension was washed thrice with 0.5 ml PBS and fixed in ice-cold 70% ethanol for 2 h at −20°C. The cells were flushed through a 21-gauge needle (3–4 times) for uniform dispersion. The fixed cells were again washed twice with PBS and incubated with PI (20 µg/ml) and RNase A (200 µg/ml) for 60 min at 37°C. Data acquisition was performed with a fluorescence-activated cell analyzer (FACS Canto, Becton-Dickinson, Franklin Lakes, NJ). For each sample, 10,000 events were acquired and the analysis was carried out using BD FACSDiva software.

### Analysis of Apoptotic Cell Death

Apoptosis was detected in single cell suspension prepared from skin of mice treated with OTA as described in the section “ROS measurement”, using Annexin V-FITC kit through flow cytometer according to the manufacturer’s protocol (BD Biosciences, San Jose, CA). Briefly, the cell pellet was suspended in 1x Annexin V-FITC binding buffer followed by incubation with Annexin V-FITC and PI in dark for 20 min. The fluorescence of the cells was analysed by flow cytometer.

### Western Blot Analysis of Proteins Involved in Oxidative Stress Signaling and Apoptotic Machinery

For western blot analysis of various proteins, the animals were distributed randomly into six groups, having three mice per group. The mice of 1^st^ group received a single topical application of 0.2 ml acetone to serve as control, while animals of 2^nd^, 3^rd^, 4^th^, 5^th^ and 6^th^ groups received a single topical application of 80 µg OTA in 0.2 ml acetone and sacrificed after 6, 12, 24, 48 and 72 h, respectively. A ∼2 cm^2^ of the exposed skin was excised from each animal and whole cell extract of mouse skin was prepared as described earlier [12]. For western blot analysis of Nrf2, nuclear extract from mouse skin was prepared as described earlier [13]. Sixty micrograms of protein lysate was resolved on 10% SDS-polyacrylamide gel and the proteins were transferred to PVDF membranes. The blotted membrane was blocked with either 5% non-fat dry milk or 5% BSA in PBS containing 0.1% Tween 20 (blocking solution), and incubated with specific antibodies against Nrf2, p-ERK1/2, p-p38, p-JNK, ERK1/2, p38, JNK, p53, p21/waf1, Bax, Bcl-2, cytochrome *c* and PARP at dilutions indicated by the manufacturer. The blots were further incubated with HRP-conjugated secondary antibody (Sigma Chemical Co., St. Louis, MO) and developed by ECL Western Blotting Detection Kit as described in the manufacturer’s protocol (Amersham, Fairfield, CT). All the blots were stripped and reprobed with β-actin to ensure equal loading of protein.

### Caspase 3, 8 and 9 Activity Assay

The treatment schedule was the same as described under the section “ROS measurement”. A ∼2 cm^2^ of the exposed skin was excised from each animal and homogenized in ice cold cell lysis buffer [10 mM Tris (pH−7.5), 10 mM NaH_2_PO_4_, 130 mM NaCl, 1% Triton X-100, 10 mM Sodium Pyrophosphate, 1 mM DTT and 250 µM PMSF] followed by incubation in ice for 30 min with intermittent vortexing to prepare protein extract of skin. The extracts were centrifuged at 12,000 g for 10 min. The supernatants were collected and 100 µg of protein extract from each sample was subjected to caspase 3, 8 and 9 activity using specific substrates conjugated with 7–amino–4–trifluoromethylcoumarin (AFC) as fluorophore (BD Pharmingen, San Jose, CA) followed by incubation at 37°C for 2 h. The fluorescence intensity was measured at an excitation and emission wavelengths of 400 nm and 505 nm, respectively, using SYNERGY-HT multiwell plate reader (Bio-Tek, Winooski, VT).

### Evaluation of Skin Tumor Initiating Potential of OTA

For tumor studies, six to seven week old female Swiss albino mice (20±3 g) were distributed randomly into three groups having ten mice per group. The following experimental protocol was adopted:

Group 1: Vehicle Control (topical application of 0.2 ml acetone treated twice weekly).

Group 2: A single topical application of DMBA (30 µg/0.2 ml of acetone) followed by twice weekly application of TPA (2.5 µg/0.2 ml of acetone) after a week of initiation.

Group 3: A single topical application of OTA (80 µg/0.2 ml of acetone) followed by twice weekly application of TPA (2.5 µg/0.2 ml of acetone) after a week of initiation.

Skin tumor formation was recorded twice weekly for 24 weeks and tumors greater than 1 mm in diameter were included in the cumulative total only if they persisted for 2 weeks or more. Cumulative tumors were defined as the total number of tumors observed on each mouse during the experimental duration.

### Histopathological Processing

The animals of tumor study were sacrificed by cervical dislocation at the termination of experiment. Skin was immediately removed, washed in cold normal saline solution, fixed in 10% buffered formalin and embedded in paraffin after processing. Skin sections of 5 µm thickness were cut and stained with hematoxylin and eosin for microscopic examination.

### Statistical Analysis

All the results were expressed as the mean ± SE. Differences between groups were analyzed using one-way ANOVA with Bonferroni intergroup comparison tests from GraphPad Prism-3.0 (GraphPad Prism software, San Diego, California, USA). A value of p<0.05 was considered as statistically significant.

## Results

### Effect of Topical Application of OTA on DNA Damage in Mouse Skin

**Figure 1:**
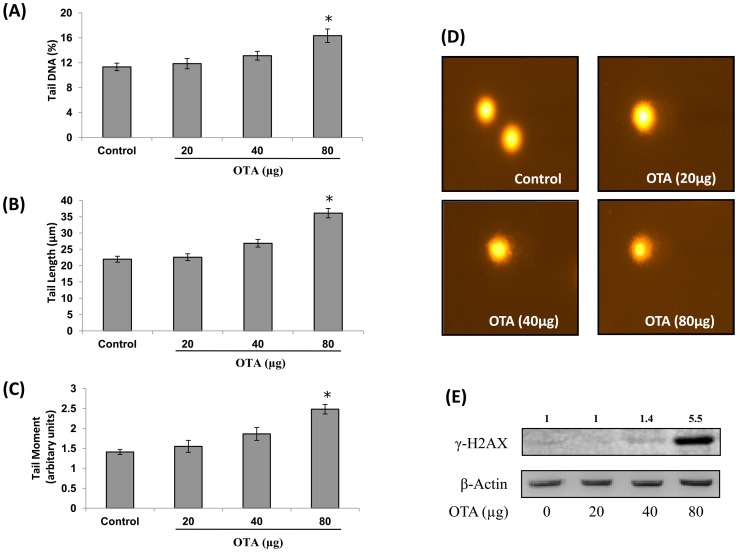
DNA damaging potential of OTA in mouse skin. Single cell suspension from vehicle or OTA (20, 40 and 80 µg/mouse) treated mouse skin was prepared and DNA damage was assessed by alkaline comet assay. DNA damage is represented in terms of (A) Tail DNA, (B) Tail Length, and (C) Tail Moment, and (D) Skin cells of vehicle treated animal without comet (400X) and OTA (80 µg/mouse) treated animals showing comet formation (400 X). Data in histogram represents mean ± SE of five animals. *p<0.05, significant with respect to control group. (E) Whole cell extract from vehicle or OTA (20, 40 and 80 µg/mouse) treated mouse skin was prepared and levels of γ-H2AX (Ser^139^) were assessed by western blot analysis. Values above the lanes of blots are mentioned as fold change with respect to control. For confirmation of equal protein loading, the blots were stripped and probed with an antibody specific for β-actin.

DNA damaging potential of OTA was evaluated in mouse skin using alkaline comet assay. As shown in Figure 1A–C, OTA treatment (20, 40 and 80 µg/mouse) caused enhancement in Olive tail moment (OTM) (110–176%), tail DNA (104–144%) and tail length (102–164%) when compared to control. The DNA from skin cells prepared from OTA treated animals showed typical formation of comet, while skin cells of control animals showed no DNA damage and had well defined circular nucleus (Figure 1D). To further confirm the DNA damaging potential of OTA, the levels of γ-H2AX (Ser^139^) were estimated in mouse skin exposed to different doses of OTA (20, 40 and 80 µg/mouse) for 24 h. Western blot analysis showed that the level of γ-H2AX was significantly elevated (5.5 fold) only at the higher dose of OTA (80 µg/mouse) (Figure 1E). OTA at the doses of 20 and 40 µg/mouse caused no significant effect on γ-H2AX levels in mouse skin.

### Effect of Topical Application of OTA on Oxidative Stress Markers in Mouse Skin

**Figure 2:**
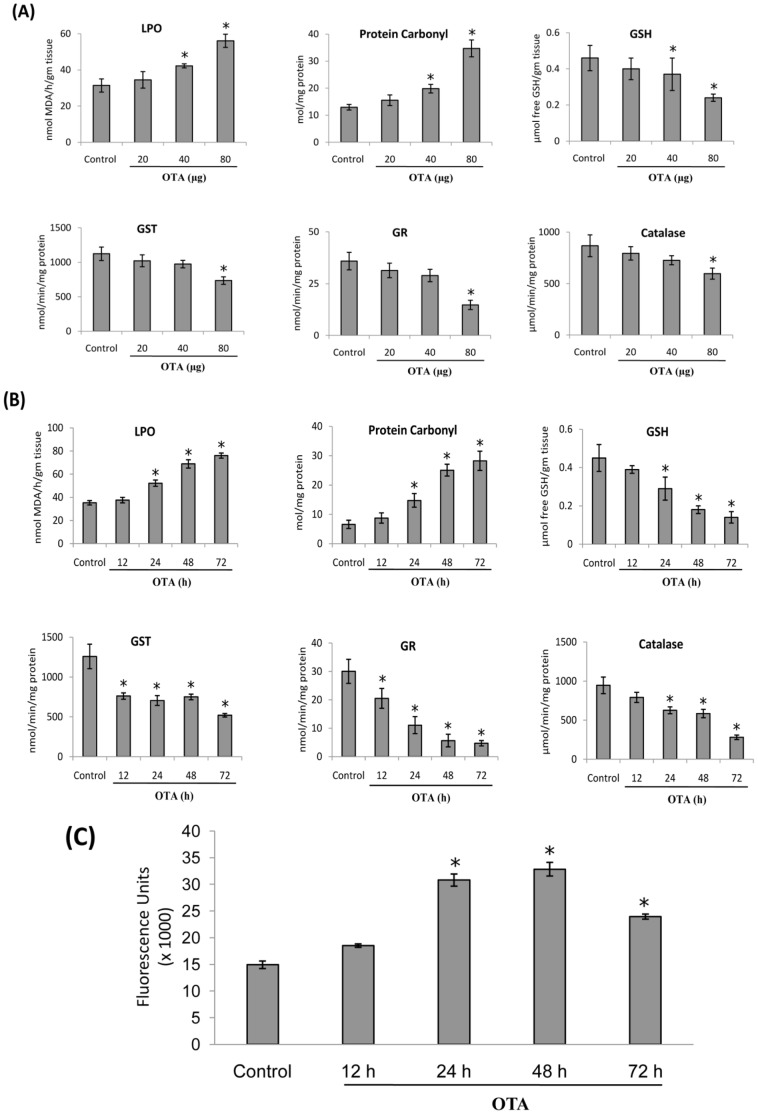
Effect of topical application of OTA on oxidative stress in mouse skin. (A) Dose dependent effect of OTA on epidermal LPO, Protein Carbonyl, GSH content, and activities of Catalase, GST and GR in mouse skin exposed for 24 h. (B) Time dependent effect of OTA (80 µg/mouse) on epidermal LPO, Protein Carbonyl, GSH content, and activities of Catalase, GST and GR in mouse skin exposed for 12–72 h. (C) Time dependent effect of OTA (80 µg/mouse) on intracellular ROS generation in mouse skin exposed for 12–72 h. Single cell suspension from vehicle or OTA (80 µg/mouse) treated mouse skin was prepared and 1×10^5^ cells were incubated with 100 µM H_2_DCFDA for 90 min at 37°C and relative fluorescence intensity was determined by spectrofluorimeter (λ_ex = _485 nm, λ_em = _520 nm). Data are represented in terms of fluorescence intensity (arbitrary units). Each value represents mean ± S.E. of five animals. *p<0.05, significant with respect to control group.

To observe the dose-dependent effect of OTA on oxidative stress markers in mouse skin, animals were treated with a single topical application of OTA (20, 40 and 80 µg/mouse) for 24 h. It was observed that OTA at the doses of 40 and 80 µg/mouse caused significant increase in LPO (34–78%) and protein carbonyl content (53–168%) along with significant decrease in GSH content (19–47%) (Figure 2A), while, the activities of catalase (31%), GR (58%) and GST (34%) were found to be significantly inhibited only at the dose of 80 µg OTA/mouse. OTA at the dose of 20 µg/mouse caused no significant effect in any of the above mentioned parameters (Figure 2A). Furthermore, to examine the time-dependent effect, mice were topically treated with OTA (80 µg/mouse) for 12, 24, 48 and 72 h. The results showed a time-dependent increase in LPO (9–121%) and protein carbonyl content (32–328%) along with significant decrease in GSH content (13–68%) and inhibition of catalase (16–70%), GR (31–84%) and GST (39–58%) activities (Figure 2B).

Since, ROS are involved in the chain reaction of LPO, the levels of ROS in OTA-treated mouse skin was measured using H_2_DCFDA as a probe. As shown in Figure 2C, topical application of OTA (80 µg/mouse) caused a significant enhancement of ROS generation in mouse skin at 24–72 h, with the maximum increase at 48 h.

### Effect of Topical Application of OTA on Nuclear Nrf2 Level and Phosphorylation of ERK1/2, p-38 and JNK MAP Kinases

**Figure 3:**
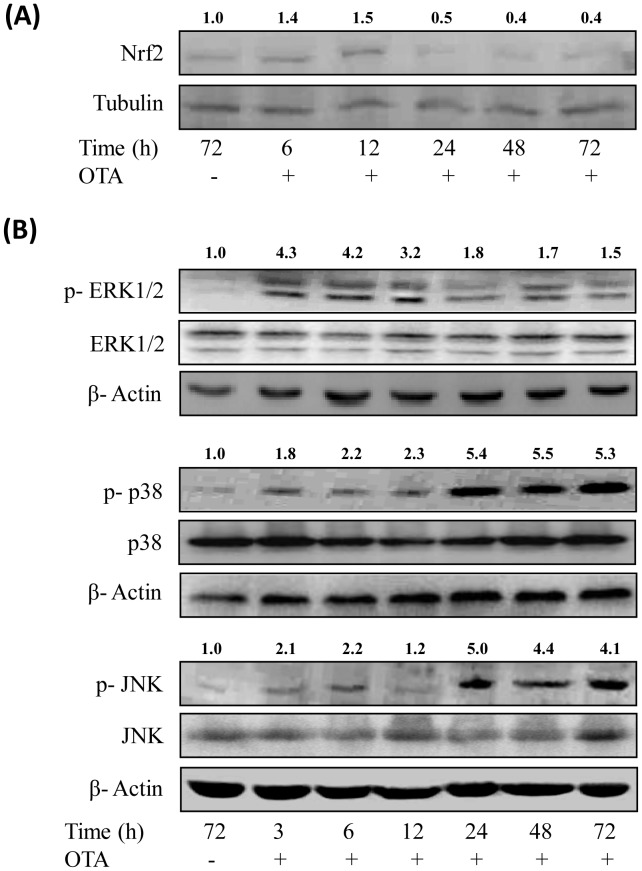
Effect of topical application of OTA on the translocation of Nrf2 transcription factor and phosphorylation of MAPKs proteins in mouse skin. (A) Nuclear extract was prepared from vehicle or OTA (80 µg/mouse) treated mouse skin and nuclear Nrf2 level was analysed by western blot analysis. Values above the lanes of blots are mentioned as fold change with respect to control. (B) Whole cell extract was prepared from vehicle or OTA (80 µg/mouse) treated mouse skin and p-ERK1/2 (Thr^202^/Tyr^204^), p-p38 (Thr^180^/Tyr^182^) and p-JNK (Thr^183^/Tyr^185^) levels and their respective total forms were analysed by western blot analysis. The values mentioned on each lane of blot represent the fold change of ratio of phosphorylated/total form with respect to control. For confirmation of equal protein loading, the blots were stripped and probed with an antibody specific for either tubulin or β-actin.

Since, Nrf2 transcription factor plays a significant role in counteracting stress conditions by inducing the expression of many genes involved in anti-oxidant responses; we studied the effect of OTA on nuclear Nrf2 levels. As shown in Figure 3A, OTA application resulted in enhancement of nuclear Nrf2 levels in mouse skin after 6 and 12 h (1.4–1.5 fold) but levels were decreased substantially after 24 h onwards.

ERK1/2, p38 and JNK are subgroup of a superfamily of serine/threonine kinases known as Mitogen-activated protein kinases (MAPKs). Due to their pivotal role in cell survival and death, the phosphorylation of ERK1/2, p38 and JNK was observed in mouse skin after OTA application. As shown in Figure 3B, a single topical application of OTA (80 µg/mouse) resulted in enhanced phosphorylation of all the three MAPKs. The level of p-ERK1/2 (Thr^202^/Tyr^204^) was enhanced at an early time point of 3 h that persisted for 12 h and then gradually subsided there after. The levels of p-p38 (Thr^180^/Tyr^182^) and p-JNK (Thr^183^/Tyr^185^) in mouse skin were significantly enhanced at 24 h following OTA application, which remained enhanced up to 72 h. However, no significant changes in the levels of total ERK1/2, p38 and JNK were observed (Figure 3B).

### Effect of Topical Application of OTA on Cell Cycle Phase Distribution and Apoptosis

**Figure 4:**
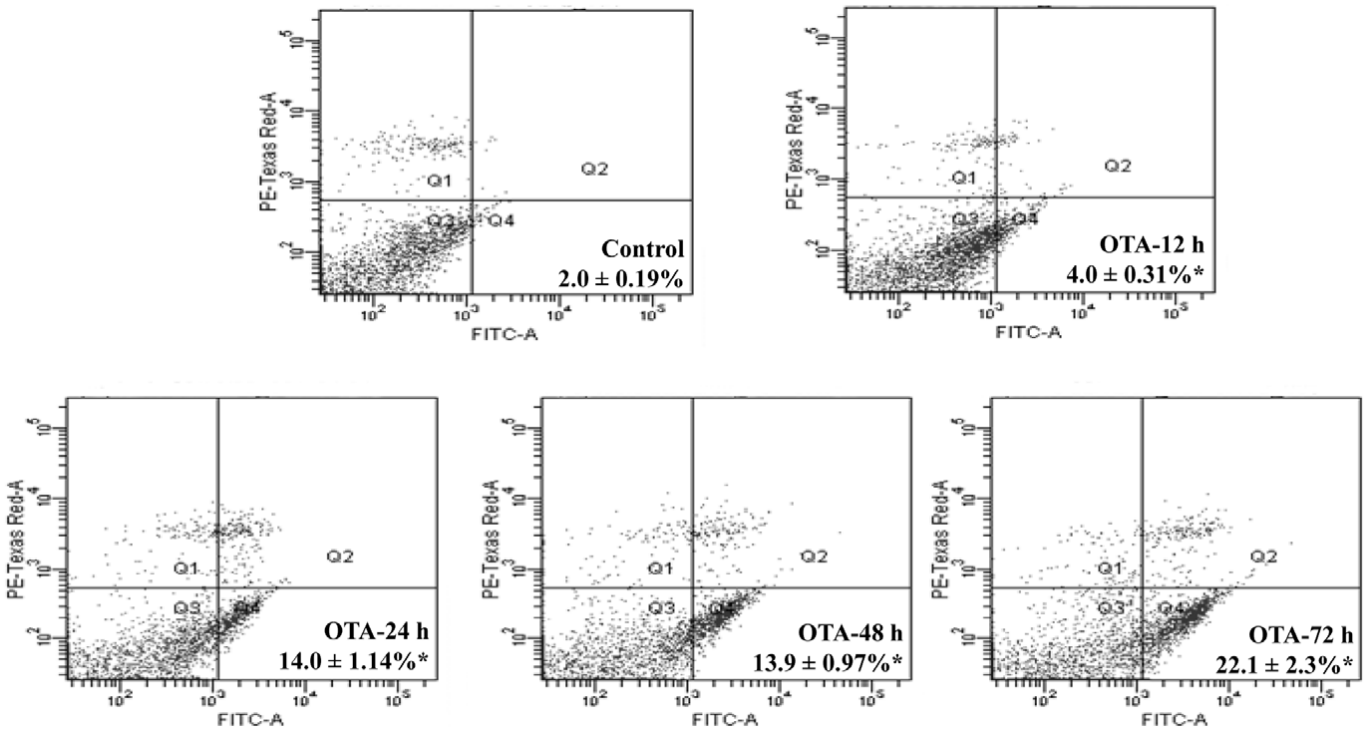
Effect of topical application of OTA on apoptosis of mouse skin cells. The single cell suspension was prepared from vehicle or OTA (80 µg/mouse) treated mouse skin for 12–72 h and apoptosis was detected by Annexin-V FITC kit through flow cytometry. Each dot plot represents 10,000 events (cells). Each value represents mean ± S.E. of five animals. *p<0.05, significant with respect to control group.

As DNA damage may lead to cell cycle arrest and apoptosis, we further analysed the cell cycle phase distribution and apoptosis after topical application of OTA. The results showed that OTA (80 µg/mouse) exposure for 24, 48 and 72 h to skin resulted in significant increase in the proportion of cells in G0/G1 phase (37–67%) with concomitant decrease in S-phase (48–63%) when compared to control group (Table 1). However, G2/M phase was not found to be significantly affected by exposure to OTA at all the tested time points. Further, it was also observed that mice treated with OTA (80 µg/mouse) for 12, 24, 48 and 72 h caused significant enhancement in apoptosis by 2.0, 7.0, 7.0 and 11.0 fold, respectively, when compared to control group (Figure 4). These results indicate that cell cycle phase arrest in G0/G1 phase is responsible for the repair of OTA induced DNA damage but some of the cells may not be able to repair the damage and undergo apoptosis.

**Table 1:** Different cell cycle phases of epidermal cells of mice topically treated with OTA (80 µg/mouse).

Group	Cell Cycle Phases		
	G0/G1	S	G2/M
Untreated	35.73±0.8	2.74±0.1	1.26±0.09
OTA (12 h)	38.55±2.0	2.57±0.07	1.33±0.03
OTA (24 h)	49.18±0.9* (37% ↑)	1.43±0.05* (48% ↓)	1.14±0.04
OTA (48 h)	54.39±0.5* (52% ↑)	1.31±0.03* (52% ↓)	1.01±0.04
OTA (72 h)	59.79±0.4* (67% ↑)	1.02±0.05* (63% ↓)	1.21±0.06

Data represent mean ± SE of five animals. Details of treatment schedule and processing of cells are described in the Materials and Methods.

Values in parenthesis indicate percent increase (↑) or decrease (↓).

* p<0.05, significant when compared with control.

### Effect of Topical Application of OTA on Expression of p53 and p21/Waf1 Proteins and Activation of Intrinsic Pathway of Apoptosis

**Figure 5:**
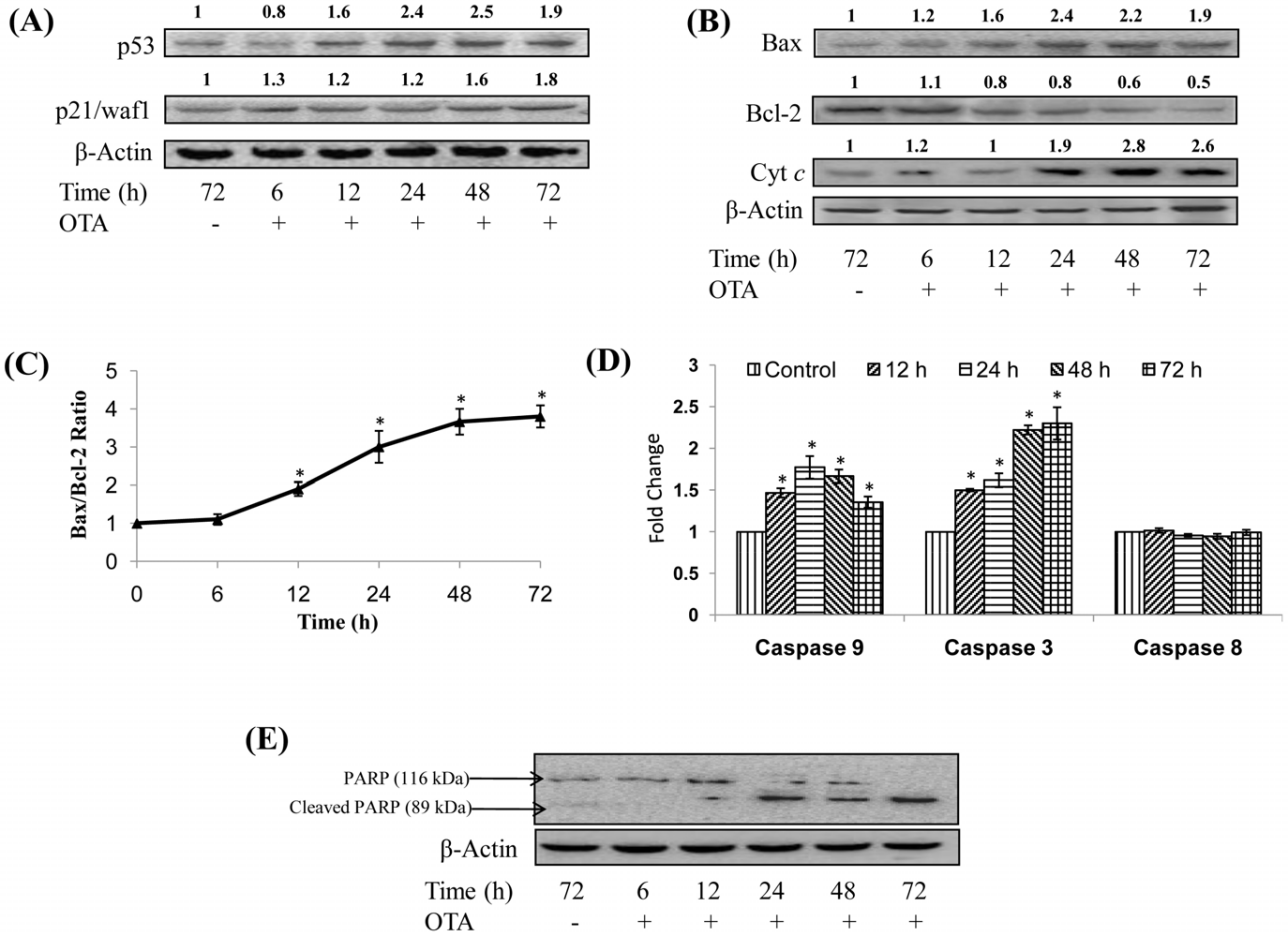
Effect of topical application of OTA on apoptotic biochemical parameters in mouse skin. (A) Whole cell extract was prepared from vehicle or OTA (80 µg/mouse) treated mouse skin and p53 and p21/waf1 levels were analysed by western blot analysis. Values above the lanes of blots are mentioned as fold change with respect to control. (B) Bax, Bcl-2 and cytochrome *c* (cyt *c*) levels in mouse skin following OTA (80 µg/mouse) exposure were analysed by western blot analysis. Values above the lanes of blots are mentioned as fold change with respect to control. (C) Bax/Bcl-2 ratio calculated on the basis of densitometric analysis of respective western blots shown in (B), (D) Caspase 3, 8 and 9 activity in mouse skin treated with OTA (80 µg/mouse) was measured using specific substrate in terms of relative fluorescence unit, at an excitation and emission wavelengths of 400 and 505 nm, respectively. Data represents mean ± S.E. of five animals and depicted as fold change as compared to control. *p<0.05, significant with respect to control, (E) Cleavage of PARP following OTA (80 µg/mouse) exposure was analysed by western blot analysis. For confirmation of equal protein loading, the blots were stripped and probed with an antibody specific for β-actin.

The effect of a single topical application of OTA (80 µg/mouse) on expression of p53 and p21/waf1 proteins in mouse skin is shown in Figure 5A. Expression of p53 was found to be significantly enhanced (1.6–2.5 fold) following OTA treatment for 12–72 h when compared to control group, whereas, p21/waf1 protein was found to be significantly over expressed (1.6–1.8 fold) between 48–72 h. Furthermore, OTA application to mouse skin for 12–72 h resulted in a significant overexpression of Bax (1.6–2.4 fold) and suppression of Bcl-2 protein levels (0.8-0.5 fold) (Figure 5B) leading to an increase in Bax/Bcl-2 ratio (1.9–3.8 fold) in mouse skin (Figure 5C). An increase in cytochrome *c* (Cyt *c*) level (1.9–2.8 fold) was also observed between 24–72 h following OTA application (Figure 5B). Further, topical application of OTA to mice resulted in significant enhancement in the activities of caspase 9 (1.2–1.8 fold) and caspase 3 (1.7–2.2 fold), which were maximum at 24 h, however, no effects were noticed in caspase 8 activity (Figure 5D). In continuation to caspase 3 activation, PARP cleavage was also observed following OTA exposure to mouse skin for 12–72 h (Figure 5E).

### Evaluation of Skin Tumor Initiating Potential of OTA

**Figure 6:**
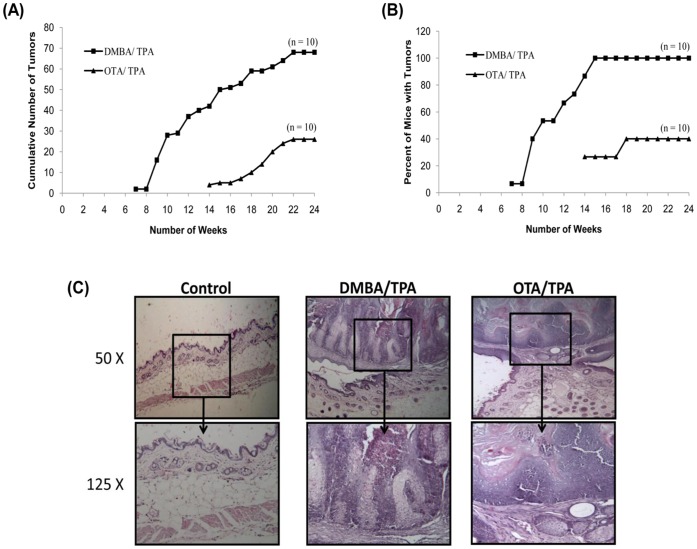
Evaluation of skin tumor initiating potential of topically applied OTA in Swiss albino mice: Comparison with single application of DMBA followed by twice weekly application of TPA to animals. Tumor incidence in DMBA/TPA and OTA/TPA groups is represented in terms of (A) Cumulative number of tumors recorded weekly, and (B) Percent of mice with tumors. No tumors were formed in the vehicle treated control group. (C) Photomicrograph of mouse skin showing normal histological structure in control animals, whereas, squamous cell carcinoma with proliferation of epidermal layers is shown in the DMBA/TPA and OTA/TPA treated groups (H&E, 50X and 125X).

Since DNA damage was observed in mouse skin following a single topical application of OTA, it was pertinent to evaluate skin tumor initiating potential of OTA in mice. A single topical application of OTA (80 µg/mouse) or DMBA (30 µg/mouse) as initiator followed by twice weekly application of TPA (2.5 µg/mouse) as promoter, resulted in the development of skin papillomas (Figure 6), whereas, no tumors were found in the vehicle treated control group. The first incidence of tumorigenesis in the group treated with DMBA (30 µg/mouse)/TPA occurred in the 7^th^ week, whereas tumorigenesis in OTA (80 µg/mouse)/TPA treated group occurred in the 14^th^ week of experiment (Figure 6A). The cumulative number of tumors in DMBA/TPA and OTA/TPA groups were 65 and 30, respectively after 24 weeks of exposure (Figure 6A). The weekly data of percent mice with tumors during the treatment schedule is presented in Figure 6B. Hundred percent mice developed tumors in DMBA/TPA treated group by the 15^th^ week of treatment. However, 40% of mice developed tumors in OTA/TPA treated group after the 18^th^ week and remained constant till the completion of 24^th^ week of treatment (Figure 6B).

Figure 6C depicts the histopathological features of skin isolated from control, DMBA/TPA, and OTA/TPA treated mouse. Control mice showed normal appearance of epidermis, dermis and hypodermis of skin. The underlying muscle layer was also visible in the control animals. In the positive control group, where animals were treated with DMBA/TPA, characteristic skin lesions including papillomatous growth comprising of squamous cell carcinoma and pearl formation was observed. The skin also showed hyperkeratosis along with hyperplasia of squamous cells protruding out of the epidermis. The skin of mice treated with OTA/TPA demonstrated circumscribed exophytic growth comprising of epithelium and supportive connective tissue extending above the epidermis. The skin from animals of OTA/TPA treated group also showed hyperkeratosis along with hyperplasia and pearl formation and at places mixed cells infiltration was evident (Figure 6C).

## Discussion

OTA, a mycotoxin, has been implicated in Balkan Endemic Nephropathy (BEN) and in the onset of urinary tract tumors in affected population, however, the mechanism of OTA induced tumorigenicity is not well understood [3], [4], [22]. Tumorigenesis is a multistage process enumerated by initiation, promotion and progression steps and it is implicated that identification of the step where OTA interferes with this process would be of significance in understanding the risk associated with its exposure [4], [6]. Although, oral exposure to mycotoxins is the common route, it has been suggested by WHO that because of employment of manual labour during pre- and post-harvest stages of agriculture, dermal exposure to these chemicals may also occur [10]. Therefore, two-stage mouse skin tumorigenesis model has been employed to study the tumorigenic property of OTA and related molecular events [23].

It has been well established that DNA damage is an important event in initiation of chemical carcinogenesis [24]. Although, there is no evidence of OTA-DNA adduct formation as metabolism of OTA fails to generate electrophilic intermediates, nonetheless, DNA damaging potential of OTA has been reported by various investigators both *in vivo* and *in vitro* [4], [7], [9], [25], [26]. Therefore, it has been suggested that ROS and oxidative DNA damage could be one of the causative factors for OTA induced toxicity and tumorigenicity [27]. In the present study, it was observed that a single topical application of OTA significantly enhanced the ROS levels along with induction of DNA damage as assessed by alkaline comet assay and increased levels of γ-H2AX protein in mouse skin. H2AX is a subfamily of the histone protein family H2A, and is found to be phosphorylated on residue serine 139 in cells when double strand breaks (DSBs) are introduced into the DNA by endogenous or exogenous factors [28]. This phosphorylation of H2AX in response to DSBs plays an important role in unwinding of nucleosomes and facilitates the recruitment of repair factors to nuclear foci [29]. Therefore, γ-H2AX is considered as a sensitive marker of DSBs or DNA damage and the observed increase in the present study indicates DNA damaging potential of OTA.

It is well known that cells have developed adaptive, dynamic programs to create a balance between production and removal of ROS, and Nrf2 transcription factor has been identified as the master regulator for maintaining the balance of ROS [30]. The present study showed that the level of Nrf2 in nucleus decreases after 24 h of OTA exposure indicating its inhibitory effect on Nrf2 signaling. In this regard studies of Cavin *et al.* [31] has suggested that OTA exposure leads to intracellular oxidative stress and DNA damage in primary rat hepatocytes and NRK cells by inhibiting the Nrf2 transcription factor. The present study demonstrated that OTA induced Nrf2 suppression may cause significant depletion in GSH content as well as inhibition in the activities of catalase, GST and GR, along with enhanced production of lipid peroxides and protein carbonyls indicating increased generation of ROS and thus enhanced oxidative stress in mouse skin.

The major group of signaling molecules which are modulated by ROS is MAPKs [32]–[34]. MAPK is a superfamily of serine/threonine kinases which are activated by many exogenous or endogenous stimuli and play a pivotal role in cell survival and death. There are three major MAPKs identified in mammalian cells: ERK1/2, p38 and JNK [35]. ERK1/2 gets activated mainly in response to growth and differentiation factors and is involved in cell proliferation, whereas, p38 and JNK MAPKs, often referred to as stress kinases, are reported to be activated in response to various environmental stresses and regulate apoptotic machinery [36], [37]. Our findings suggest that OTA activates ERK1/2, p38 and JNK MAPKs at the different time points. ERK1/2 was found to be activated at an early time point of 3 h with maximum increase at 6 h and gradually subsided afterwards, whereas, p38 and JNK were found to be significantly activated at 24 h and remained activated up to 72 h. The early activation of ERK1/2 observed in the present study may be the result of direct response to OTA but the late activation of p38 and JNK at 24–72 h coincides with the enhanced formation of ROS indicating that OTA induced ROS may acts as secondary messenger in intracellular signaling cascade by activating these two stress kinases in mouse skin [34]. Moreover, it has been reported that mere activation of p38 and JNK may not be necessary for apoptosis under all conditions, but concurrent inhibition of ERK1/2 is also a critical factor [38]. Thus, a dynamic balance between ERK pathway and p38 and JNK pathways determines whether a cell will survive or undergo apoptosis [39] which has been observed to be shifting towards p38 and JNK, leading to apoptosis in mouse skin following dermal exposure to OTA.

Apoptosis plays a vital role in multi cellular organisms by eliminating unwanted and/or damaged cells. It has been suggested that numerous chemical and physical treatments which stimulate oxidative stress via ROS generation and subsequent DNA damage, are capable of inducing apoptosis [40]. The present findings suggest that exposure to OTA results in a significant increase in the proportion of cells in G0/G1 phase with a concomitant decrease in S-phase followed by increase in apoptosis in mouse skin. This may be due to the fact that the cells respond to DNA damage by arresting the cell cycle progression to repair the damage [41]. However, if DNA damage is irreparable, the cells may undergo apoptosis as observed in the present study. In support to these findings, several *in vivo* and *in vitro* studies have shown that OTA caused oxidative DNA damage in kidney, liver and cell systems leading to apoptosis [26], [31], [42], [43]. Furthermore, Mantle et al. have also suggested that apoptosis might play a role in OTA induced nephropathy [44].

The process of cell cycle arrest and apoptosis in response to DNA damage is primarily mediated by tumor suppressor protein, p53 [41], [45]. Our results suggest that topical application of OTA causes DNA damage resulting in overexpression of p53 protein, thereby up regulating its target protein, p21/waf1, in mouse skin. The up regulation of p21/waf1 protein results in inhibition of Cyclin D1/Cdk complex causing cell cycle arrest [46]. p53 protein along with cell cycle checkpoint machinery regulate the levels of various proteins of Bcl-2 family; and thus act as the master regulator of apoptosis via intrinsic pathway [45], [47]. The present study showed that topical application of OTA resulted in up regulation of Bax (pro-apoptotic protein) and down regulation of Bcl-2 (anti-apoptotic protein), thus shifting the balance towards pro-apoptotic signal resulting in increased levels of cytochrome *c.* The OTA induced cytochrome *c* leads to auto-activation of caspase 9, which further activates caspase 3. The executor, caspase 3 induced by OTA finally cleaves PARP protein, a DNA repair enzyme, leading to apoptosis in mouse skin cells [47]–[49]. Furthermore, the observed lack of enhancement in caspase 8 activity indicates that the extrinsic or death receptor pathway is not involved in OTA induced apoptosis in mouse skin. Earlier studies have shown that OTA induced apoptosis in various cell lines was mediated through the alteration in the homeostasis between Bcl-2 family proteins, thereby inducing mitochondria dependent apoptosis which also corroborates our *in vivo* findings [50], [51].

Several studies involving chemical carcinogenesis in animal models clearly suggest a close correlation between chemical exposure, DNA damage, mutagenesis and tumor formation [24]. The observed ROS generation and DNA damage by OTA in mouse skin indicates the tumor initiating potential of OTA. The present study also showed that OTA when tested as tumor initiator in a two stage mouse skin tumorigenesis protocol resulted in tumor formation. OTA showed all the characteristic features of a tumor initiator similar to that of DMBA, a well known skin tumor initiator having DNA damaging and oxidative stress capability [52]–[54]. The tumor initiating dose of OTA (80 µg/mouse) used in the present study finds significance as OTA contamination in various food crops has been detected at the levels of 3800 µg/kg in barley sample from Czechoslovakia, 2400 µg/kg in rye sample, 2400 µg/kg in wheat sample from Poland, and 166 µg/kg in corn sample from US [55]–[58]. Thus, it is quite likely that humans involved in agricultural practices may get OTA exposure through skin to elicit dermal effects.

Our results suggest that a single topical application of OTA caused DNA damage along with overexpression of wild-type p53 and subsequently cell cycle arrest and apoptosis in mouse skin. It is quite likely that some cells may pass through p53-mediated cell cycle checkpoint by faulty repair which may introduce mutations in OTA-induced animals and subsequent applications of TPA, a tumor promoter, fix the mutations and confers selective advantage to those cells which leads to tumorigenesis. In this regard, overexpression of p53 and apoptosis has been reported as an early event following exposure to known DNA damaging and skin tumor initiating carcinogens such as benzo(a)pyrene or DMBA, but this overexpression of p53 is not sufficient to inhibit carcinogenesis (53,54,59–63).

In conclusion, the present study reveals that OTA has skin tumor initiating property under *in vivo* condition, which may be related to the oxidative stress, MAPKs signaling and DNA damage in mouse skin and anti-oxidants may have a role in the prevention of OTA induced tumorigenesis which needs to be investigated.
