# Correction: Topical Application of Ochratoxin A Causes DNA Damage and Tumor Initiation in Mouse Skin

**DOI:** 10.1371/journal.pone.0208284

**Published:** 2018-11-26

**Authors:** Rahul Kumar, Kausar M. Ansari, Bhushan P. Chaudhari, Alok Dhawan, Premendra D. Dwivedi, Swantantra K. Jain, Mukul Das

Incorrect images were used for the Control and OTA (80μg) panels of Fig 1D of this article [1], which are duplicates of the Control and CTN (48h) panels, respectively, in Fig 2B of the following article previously published in the journal *Toxicological Sciences*:

Citrinin-Generated Reactive Oxygen Species Cause Cell Cycle Arrest Leading to Apoptosis via the Intrinsic Mitochondrial Pathway in Mouse Skin [2]

Author list: Rahul Kumar, Premendra D. Dwivedi, Alok Dhawan, Mukul Das, Kausar M. Ansari

The authors apologise for this inadvertent error during figure preparation.

The authors provide an updated Fig 1 here which includes data collected at the same time as the other images. The authors also provide raw data underlying Fig 1 panels A-D as Supporting Information files below.

Due to the time elapsed since experiments were conducted, data underlying Fig 1E and all other Figures in the article are no longer available.

**Fig 1:**
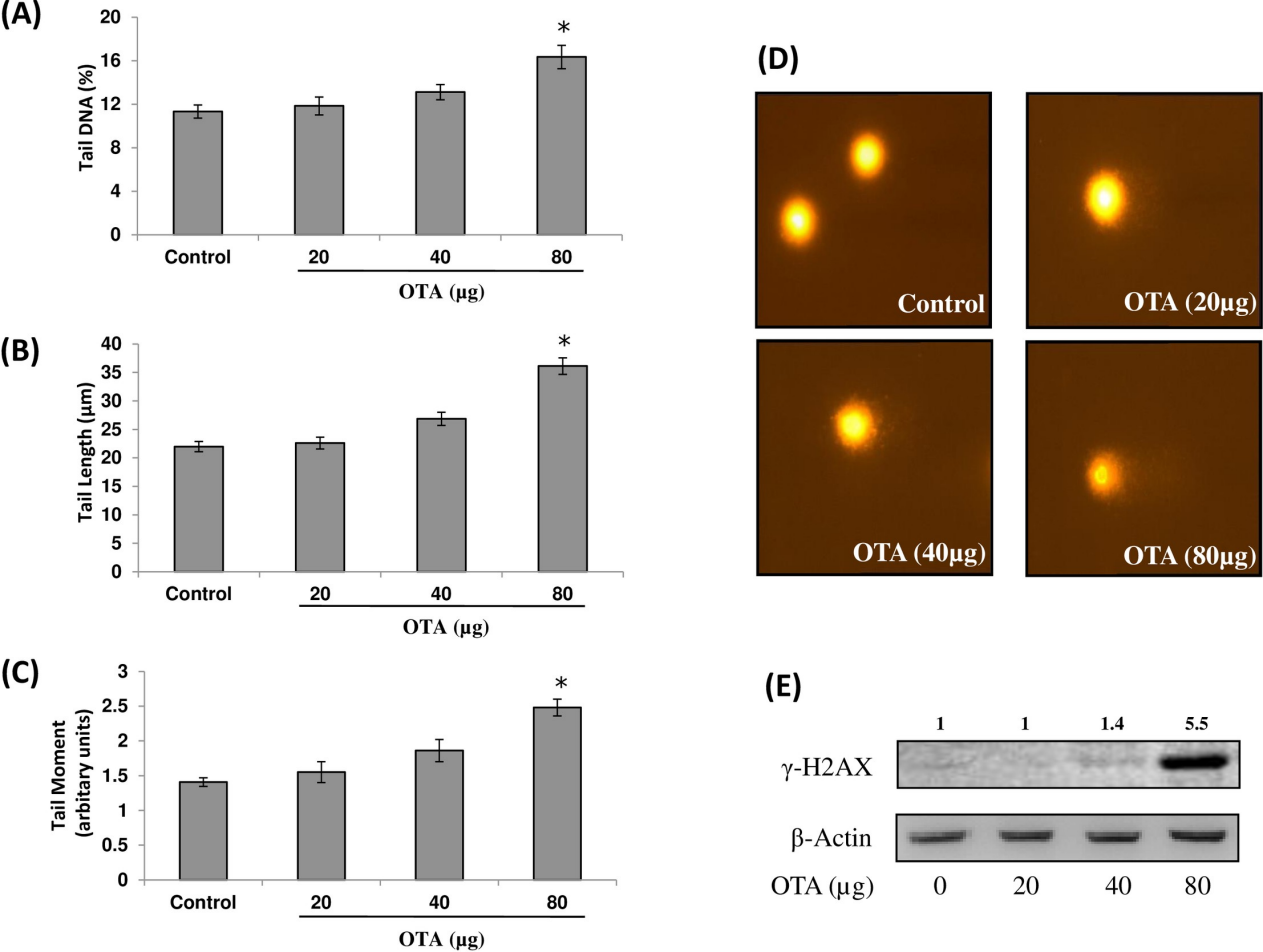
DNA damaging potential of OTA in mouse skin. Single cell suspension from vehicle or OTA (20, 40 and 80 μg/mouse) treated mouse skin was prepared and DNA damage was assessed by alkaline comet assay. DNA damage is represented in terms of (A) Tail DNA, (B) Tail Length, and (C) Tail Moment, and (D) Skin cells of vehicle treated animal without comet (400X) and OTA (80 μg/mouse) treated animals showing comet formation (400 X). Data in histogram represents mean ± SE of five animals. *p<0.05, significant with respect to control group. (E) Whole cell extract from vehicle or OTA (20, 40 and 80 μg/mouse) treated mouse skin was prepared and levels of γ-H2AX (Ser139) were assessed by western blot analysis. Values above the lanes of blots are mentioned as fold change with respect to control. For confirmation of equal protein loading, the blots were stripped and probed with an antibody specific for β-actin.

## Supporting information

S1 Data Raw data underlying Fig 1. (ZIP)
