# Supplementary figures and images for: Correction: Topical Application of Ochratoxin A Causes DNA Damage and Tumor Initiation in Mouse Skin
Source: PLoS One. 2018 Nov 26;13(11):e0208284. doi: 10.1371/journal.pone.0208284 (PMC6258368; doi:10.1371/journal.pone.0208284)

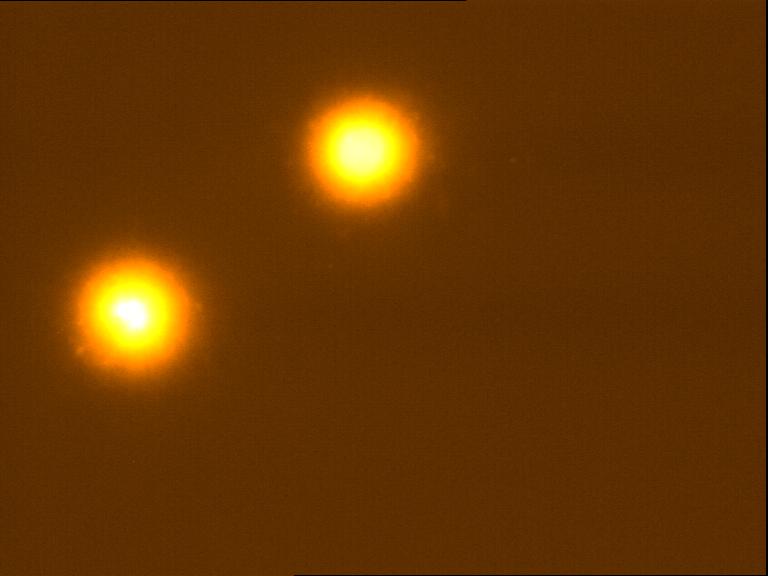

Supplement: S1 Data — (ZIP) [file pone.0208284.s001.zip › S1 Data/New Image for Revised Control.jpg]

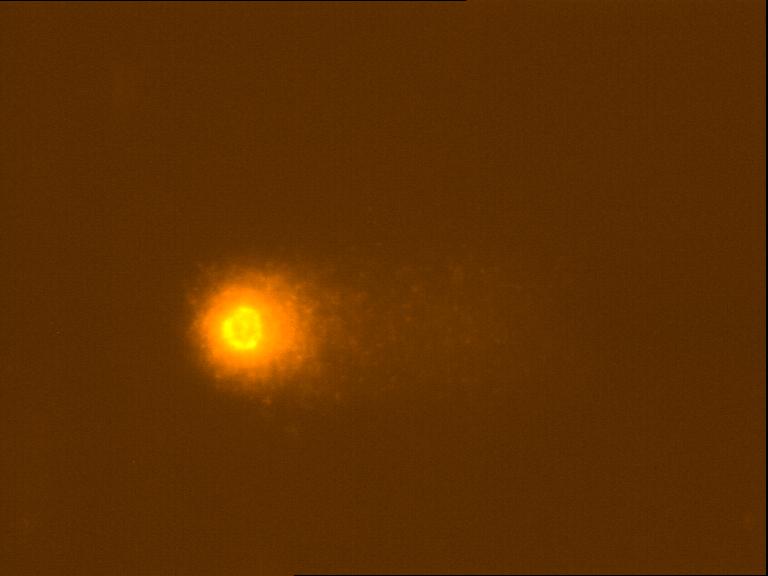

Supplement: S1 Data — (ZIP) [file pone.0208284.s001.zip › S1 Data/New Image for Revised OTA (80ug).jpg]

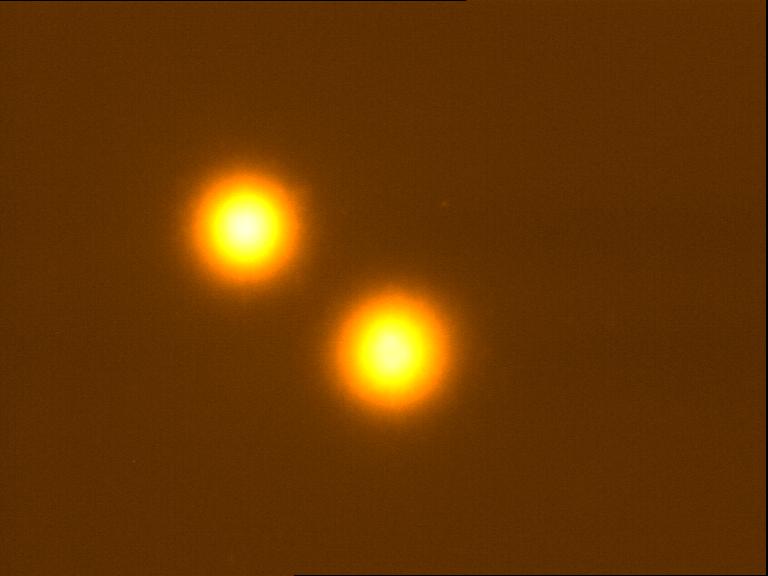

Supplement: S1 Data — (ZIP) [file pone.0208284.s001.zip › S1 Data/Old Control_Image.jpg]

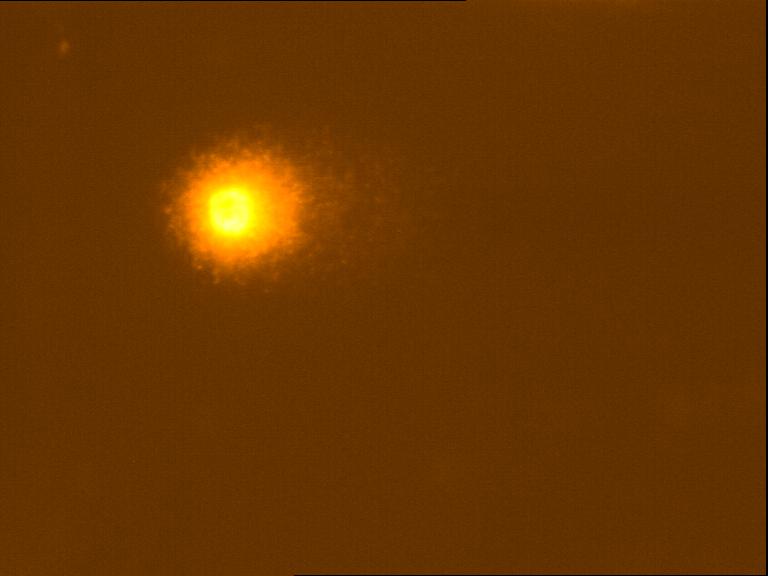

Supplement: S1 Data — (ZIP) [file pone.0208284.s001.zip › S1 Data/Old uncropped OTA (80ug)_Image.jpg]

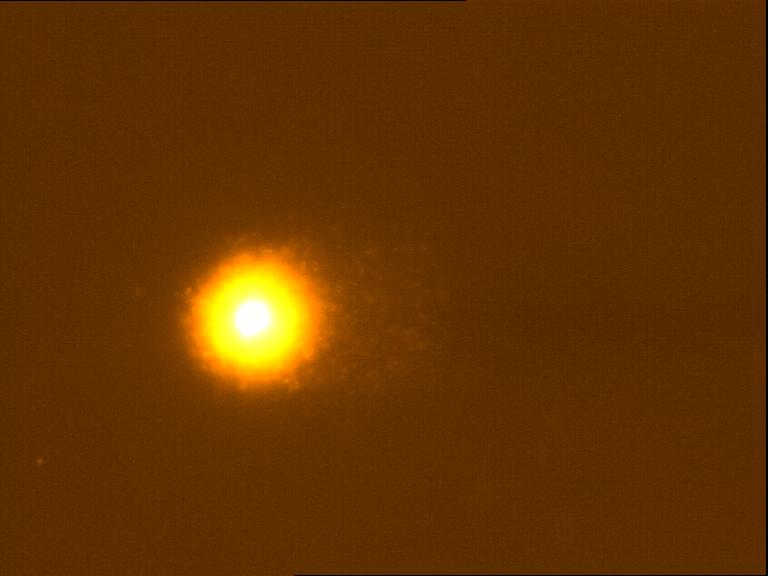

Supplement: S1 Data — (ZIP) [file pone.0208284.s001.zip › S1 Data/OTA (20ug)_Image.jpg]

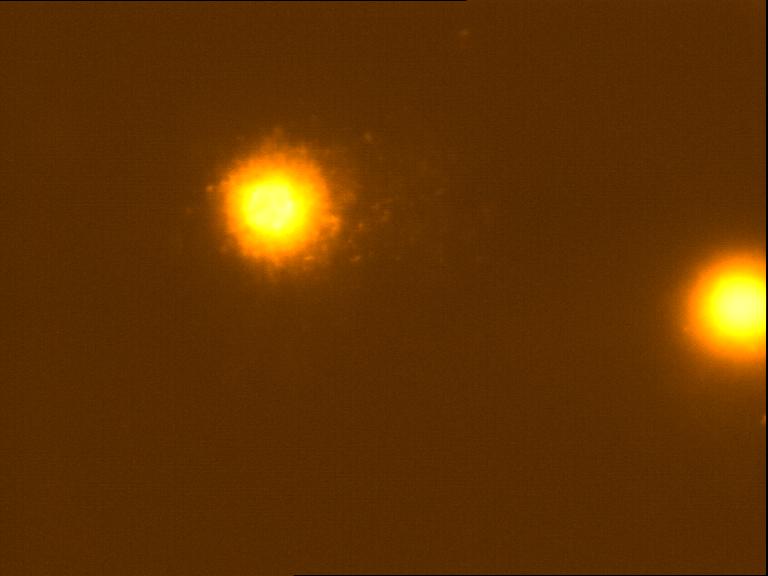

Supplement: S1 Data — (ZIP) [file pone.0208284.s001.zip › S1 Data/OTA (40ug)_Image.jpg]
